# Dietary alpha‐ketoglutarate inhibits SARS CoV‐2 infection and rescues inflamed lungs to restore O_2_ saturation by inhibiting pAkt

**DOI:** 10.1002/ctm2.1041

**Published:** 2022-09-19

**Authors:** Sakshi Agarwal, Simrandeep Kaur, Tejeswara Rao Asuru, Garima Joshi, Nishith M Shrimali, Anamika Singh, Oinam Ningthemmani Singh, Puneet Srivastva, Tripti Shrivastava, Sudhanshu Vrati, Milan Surjit, Prasenjit Guchhait

**Affiliations:** ^1^ Regional Centre for Biotechnology National Capital Region Biotech Science Cluster Faridabad India; ^2^ Translational Health Science Technology Institute National Capital Region Biotech Science Cluster Faridabad India

Dear Editor,

In order to combat the COVID‐19 pandemic, an extensive effort is being made by researchers around the world to develop therapeutics against Severe Acute Respiratory Syndrome Coronavirus‐2 (SARS CoV‐2). As the name indicates, this virus primarily affects the respiratory tract including lungs. Unlike with other respiratory viral infections, symptoms of COVID‐19 are very heterogeneous ranging from minimal to Acute Respiratory Distress Syndrome (ARDS).^1^ Elevated intravascular clots, accumulation of leukocytes including neutrophils and macrophages, deposition of collagen, mucus, and other extracellular matrix, and accumulation of fluid in alveoli lead to reduced blood‐gas barrier permeability and exchange of O_2_, causing a condition called hypoxemia or decreased oxygen pressure saturation (SpO_2_) in COVID‐19.^2^

With a focus on our investigation to inhibit viral infection and rescue lung pathogenesis in COVID‐19, we tested the effect of alpha‐ketoglutarate (αKG), a common metabolite of the Krebs cycle, in SARS CoV‐2‐infected animals. Recently, we reported that αKG, a co‐factor of prolyl hydroxylase 2(PHD2), augmented the prolyl hydroxylation activity of the enzyme and significantly degraded its substrates such as phosphorylated‐Akt (pAkt)^3^ and HIF1α / HIF2α.^4^ Dietary supplementation of αKG significantly reduced pro‐inflammatory and pro‐thrombotic responses of leukocytes and platelets in conjunction with downmodulation of pAkt in mice.^3^ Studies have reported the usage of αKG to improve human health. Administration of αKG in blood cardioplegia improved myocardial protection in patients with heart surgery.^5^ Dietary supplementation of αKG along with vitamin delayed human aging.^6^

It is now known that (1) SARS CoV‐2 employs Akt signalling for propagation in its host^7^ and (2) pAkt promotes platelet activation and thrombosis as well as leukocyte activation and inflammation in SARS CoV‐2‐infected animals.^3^ We, therefore, investigated whether (1) αKG could inhibit SARS CoV‐2 replication by downmodulating pAkt in vitro and in vivo, and (2) dietary supplementation of the metabolite could rescue the lung pathogenesis to restore normal O_2_ saturation in infected animals.

We report that supplementation of octyl‐αKG significantly inhibits SARS CoV‐2 replication in conjunction with the downmodulation of pAkt‐Ser473 and pAkt‐Thr308 in the Vero E6 cell line in vitro (Figures 1A–C and S1C) as well as in the human monocytic U937 cell line transiently expressing ACE2(Figures 1D–E, H and S2C), similar to the effect of Akt‐inhibitor, triciribine (TCN; Figures 1A–C and S1C). More importantly, octyl‐αKG supplementation did not alter the mitochondrial function and ATP release in the cells (Figure S1D). Octyl‐αKG decreased ACE2 expression (Figure 1C), potentially by inhibiting pAkt signalling.^8^ On the other hand, octyl‐αKG did not inhibit viral replication (Figures 1F and G and S2D) and pAkt expression (Figure 1J) in PHD2‐knockdown U937 cells, thus confirming that the PHD2‐pAkt is a target axis of this metabolite. Also we observed an inhibitory effect of octyl‐αKG on the other substrates of PHD2, such as HIF1α and HIF2α (Figures 1C and S3I and J). A recent study has described that SARS CoV‐2 ORF3 protein induced HIF1α to facilitate viral replication and inflammation in patients.^9^ However, a further detailed study may explore the role of αKG‐PHD2‐HIFα axis on SARS CoV‐2 replication. Although the enzyme activity of PHD2 was altered after SARS CoV‐2 infection and/or after octyl‐αKG supplementation, no change in expression of PHD2 protein was observed (Figure 1C). Besides, we also tested the effect of a non‐esterified αKG (NE‐αKG) on viral replication. Unlike the effect of a lower concentration of octyl‐αKG (0.75–1.5 mM,Figures S1C and 1A–C), the NE‐αKG showed an inhibitory effect on viral replication alongside downmodulation of pAkt at higher concentrations of 5–7.5 mM (Figure S1E and F). This could be because of low uptake of αKG by Vero cells when treated with NE‐αKG compared to octyl‐αKG (Figure S9A).

**FIGURE 1:**
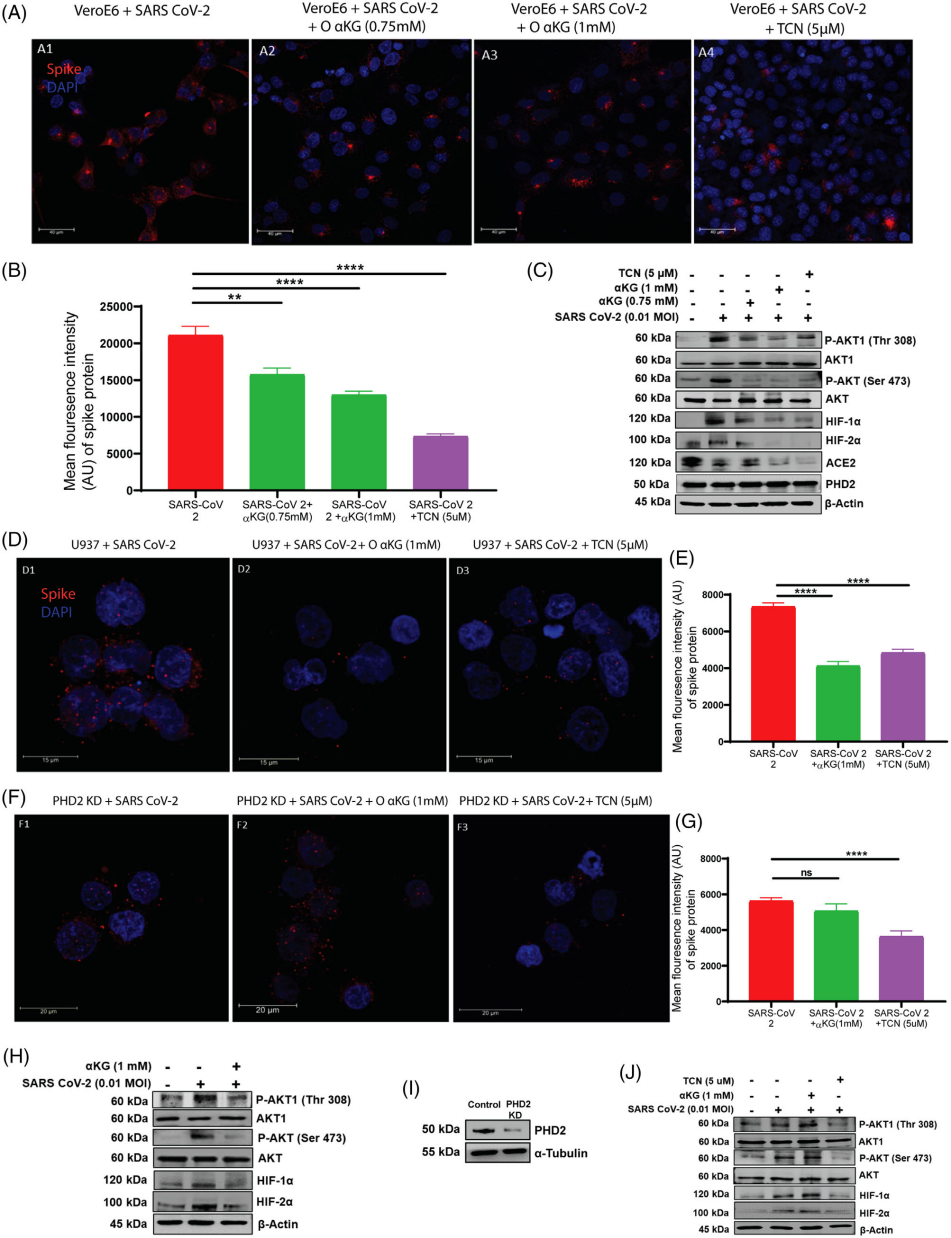
αKG inhibits SARS CoV‐2 viral replication via the pAkt‐PHD2 axis: (A) Vero E6 cells were infected with SARS CoV‐2 (0.01 MOI) for 24 h in the presence of αKG or TCN. (B) αKG decreased viral replication as measured by Spike protein quantification using confocal microscopy as represented as mean ± SEM of MFI from 50 cells from different experiments (Kruskal‐Wallis test followed by Sidak's multiple comparison post‐test), **p < .01, ****p < .0001. PCR data of the viral genome from the above experiment is described in Figure S1C. (C) Protein expression of pAkt1‐Thr308, pAkt‐Ser473, HIF1α, HIF2α, ACE2 and PHD2 was detected by western blot. The expression of all above proteins except PHD2 were increased upon viral infection and decreased by αKG or TCN. Densitometry quantification of blots is described in Figures S4A–F (Kruskal‐Wallis test followed by Dunn's multiple comparison post‐test), *p < .05, **p < .01, ***p < .001, ****p < .0001 and ns = non‐significant. (D) αKG or TCN decreased Spike protein in the U937 cell line transiently expressing hACE2 (described in Figure S2A). (F) αKG was unable to decrease Spike protein levels in PHD2‐knockdown U937 cells, but TCN decreased the same in PHD2‐KD U937 cells. (E, G) MFI is represented as mean ± SEM, ****p < .0001 and ns = non‐significant. (H) Expression of pAkt1‐Thr308, pAkt‐Ser473, HIF1α and HIF2α were decreased upon treatment with αKG in wild‐type U937 cells, (I, J) but found unaltered in PHD2‐KD U937 cells with 80%–90% knockdown efficiency (I, densitometry in Figure S4H). Densitometry quantification is described in Figures S4J–M, S4N–Q. *p < .05, **p < .01, ***p < .001, ****p < .0001 and ns = non‐significant

Here we show that oral gavage of a dietary grade αKG (40 mg/100 g body wt./day; Figure 2A) till 4 days post‐infection of SARS CoV‐2 significantly inhibited viral load (Figures 2B and S3C) and downmodulated pAkt (Figures 2I and S3G and H) and HIF2α (Figures 2I and S3I and J) in hamster lungs at 5 days post‐infection (dpi). The αKG supplementation till 4 days showed significant inhibitory effect on SARS CoV‐2 replication even at 9 dpi (Figure S3). Besides, αKG decreased infection‐induced accumulation of inflammatory cells in alveolar spaces (Figure 2C and D) and clot formation in micro vessels (Figure 2E and F), and also reduced apoptotic tissue damage (Figure 2G and H) in the infected lungs. Since, the elevated inflammation/thrombosis/apoptosis is known to cause ARDS and hypoxemia, leading to a decreased SpO_2_ in the circulation of COVID‐19 patients,^1^, ^2^ we measured circulating SpO_2_ in infected animals from the above experiment and observed a significant rescue effect of αKG in restoring normal SpO_2_ saturation at 4 dpi onwards (Figure 2J). The above experiments in balb/c mice transiently expressing hACE2 also showed a similar result. SARS CoV‐2‐infected both male (Figure S6 and S7) and female mice (Figure S8). In mice, we could assess some additional parameters showing that the αKG supplementation significantly reduced the elevated counts of leukocytes (Figure S6L and N) and leukocyte‐platelet aggregates (Figure S6M and O) along with levels of IL6, TNFα and IL10 in lungs (Figure S6S–U) as well as in plasma (Figure S7A–F) of infected mice. αKG also reduced thrombogenic markers like platelet (CD41a+) microparticles in the circulation of infected mice (Figure S7G and H), suggesting a potent anti‐thrombotic role of the metabolite. αKG supplementation improved SpO_2_ in infected mice as well (Figure S7I). As reported in our recent works,^3^, ^4^ we measured an elevated level of αKG in circulating leukocytes as well as in lung tissues of animals after αKG supplementation (Figure S9).

**FIGURE 2:**
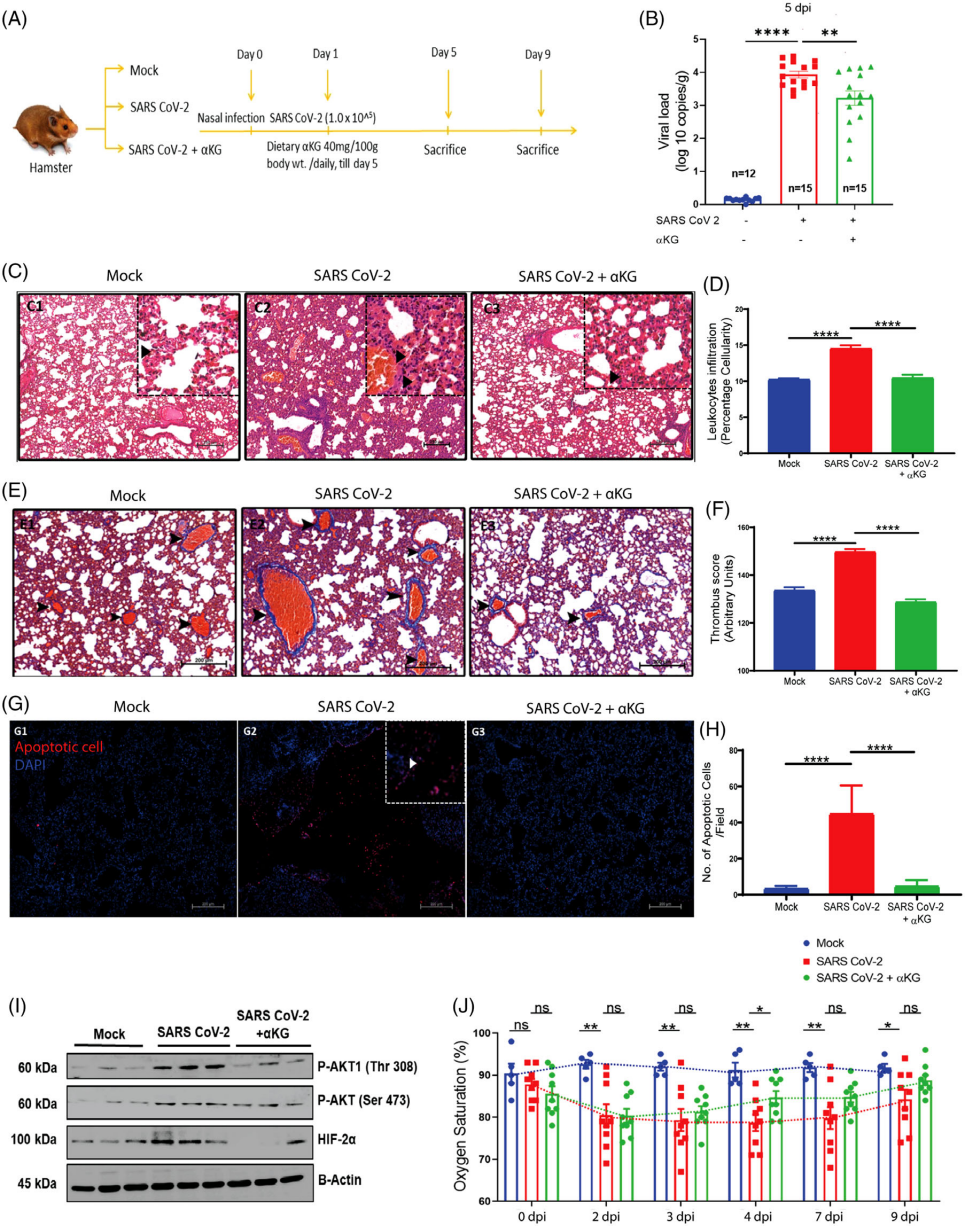
αKG rescues hamsters from SARS CoV‐2 infection and restores SpO_2_ in the circulation: (A) Hamsters were given SARS CoV‐2 infection via the nasal route and αKG was supplemented from 1 day post‐infection (dpi) and animals were sacrificed at 5 dpi and 9 dpi to measure the following parameters. (B) αKG reduced SARS CoV‐2 replication in lung at 5 dpi. Data are from 12 mock, and 15 infected and 15 infected+αKG groups. Each dot represents an individual value expressed as mean ± SEM (one‐way ANOVA using Sidak's post‐test), **p < .01 and ****p < .0001. (C, D) H&E staining of lung was used to assess leukocyte accumulation at 5 dpi. (D) Percentage cellularity score was calculated as mean ± SEM, from 10 fields from different animals and (one‐way ANOVA, using Bonferroni's post‐test **** *p* < .0001). Arrows indicate cell accumulation. Scale bar 250 mm. (E) MT staining of lung was used for assessing clot formation at 5 dpi; arrows indicate thrombus (F). Thrombus score is represented as mean ± SEM (one‐way ANOVA, using Bonferroni's post‐test *p < .05, ***p < .001 and **** *p* < .0001). Scale bar 200 mm. (G) TUNEL assay was used to assess apoptotic cells. αKG treatment reduced apoptosis in the lungs of infected hamsters at 5 dpi. (H) Quantification of apoptotic cells per field. 3X3 field captured at 20× magnification. Data are from 10 fields from different animals and represented as mean ± SEM (one‐way ANOVA, using Bonferroni's post‐test ****p < .0001) The above data for 9 dpi are described in Figure S3O and P. (I) Expression of pAkt1‐Thr308, pAkt‐Ser473 and HIF2α measured in SARS CoV‐2‐infected hamsters supplemented with αKG. Densitometry quantification from different animals is described in Figure S4R–T. (Kruskal‐Wallis test followed by Dunn's multiple comparison post‐test), *p < .05, **p < .01, ***p < .001, ****p < .0001, and ns = non‐significant. (J) Oxygen saturation in the circulation was measured in hamsters from mock (*n* = 5), infected and infected+αKG groups (*n* = 9 in each) using a pulse oximeter. Infected animals showed a significant decrease from 2 dpi till 9 dpi compared to mock. αKG showed a rescue effect from 4 dpi onwards. Data are represented as mean ± SEM (Student's *t* test, *p < .05 and ns = non‐significant)

We thereafter investigated the effect of αKG on adaptive immune parameters (as described in our recent work^10^) in hamsters. We observed similar levels of IgG against SARS CoV‐2 RBD protein in plasma between infected and infected+αKG groups at 5 dpi (Figure 3A and B) and 9 dpi (Figure 3C and D). We observed a similar efficacy of the plasma‐containing antibody from αKG supplemented animals in neutralizing the SARS CoV‐2 compared to only the virus‐infected group (Figure 3E and F), suggesting no significant interference of this metabolite on antibody response. We also investigated the anti‐viral response of T lymphocytes between groups. Dietary αKG did not alter the already elevated percentage of interferon‐γ positive (IFNγ+) CD4+ (Figure 3G and I) and IFNγ+CD8+ (Figure 3H and J) T cells in the spleen compared to the virus only at 5 dpi and 9 dpi respectively. The observations raise the possibility that the T‐cell response may be blunted following αKG treatment as it inhibits viral replication. Another study has described that αKG supplementation activates Th1 cell function.^11^ A further detailed investigation may explain the role of αKG on T and B cell responses in SARS CoV‐2 infection. However, our data strongly suggest a safe usage of the metabolite without suppressing the anti‐viral response of these adaptive immune cells.

**FIGURE 3:**
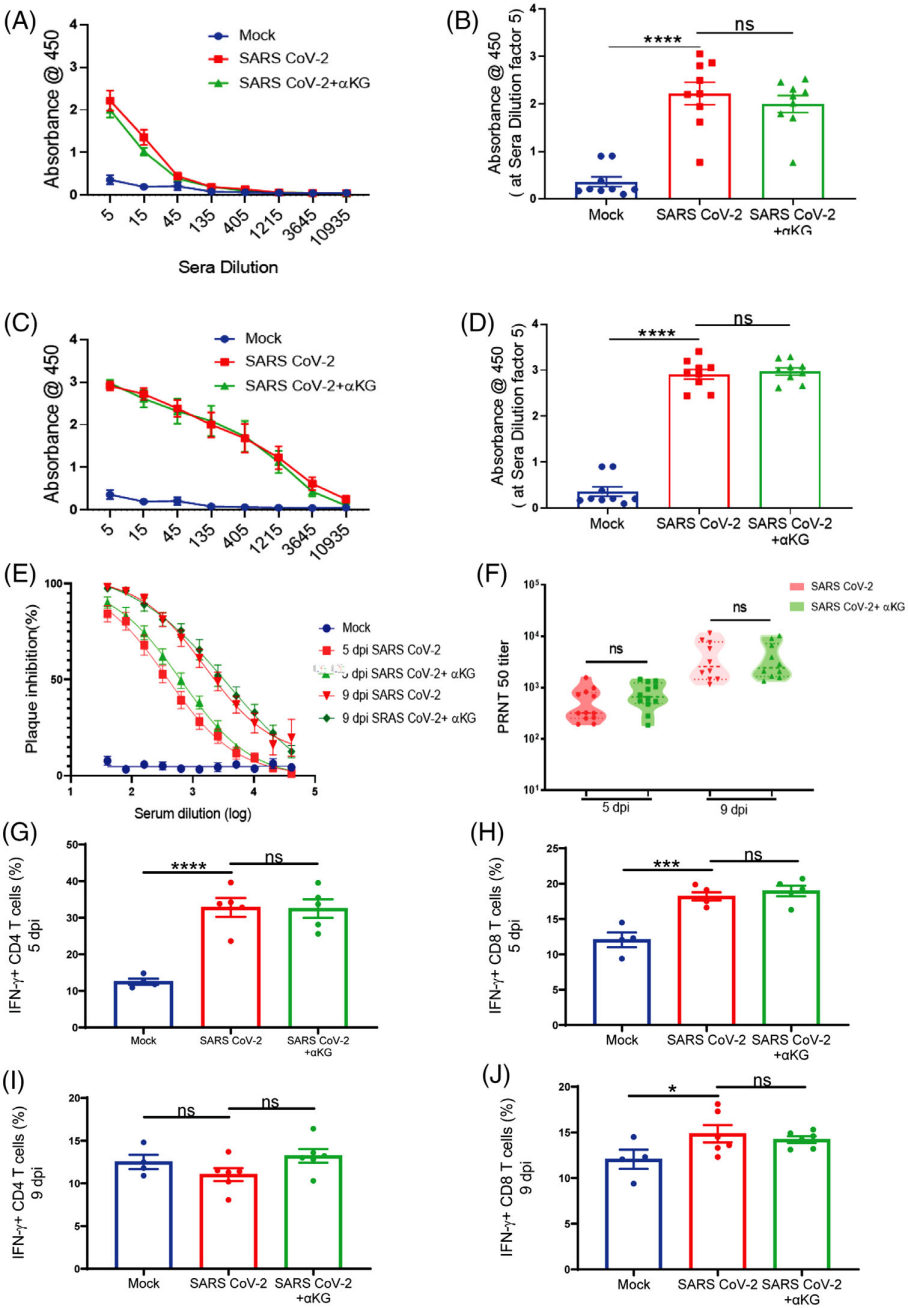
αKG supplementation does not interfere with the anti‐viral response of T cells and IgG in SARS CoV‐2‐infected hamsters. Anti‐SARS RBD antibody quantification at increasing sera dilution showed no difference between infected and infected+αKG groups at 5 dpi (A) and 9 dpi (C). Absorbance at sera dilution 5 at 5 dpi (B) and 9 dpi (D). Data from 9 animals in each group are represented as mean ± SEM (one‐way ANOVA, using Bonferroni's post‐test, ****p < .0001 and ns = non‐significant). (E, F) Neutralization antibody was measured at increasing sera dilution using a PRNT_50_ assay showing no difference between the above groups at 5 dpi and 9 dpi. Data are represented as mean ± SEM (*n* = 12 in each group) at 5 dpi and (*n* = 10 in each group) at 9 dpi (one‐way ANOVA, ns = non‐significant). Flow cytometry analysis of IFNγ+ CD4 T‐cell percentage from spleen showing no difference between infected and infected+αKG groups at 5 dpi (G) and 9 dpi (I). Data are represented as mean ± SEM from 4 animals in mock, and 5 in infected and 5 in infected+αKG groups at 5 dpi, and 6 animals in infected and 6 in infected+αKG groups at 9 dpi (one‐way ANOVA, using Bonferroni's post‐test, ****p < .0001 and ns = non‐significant). Analysis of IFNγ+ CD8 T‐cell percentage showing no difference between groups at 5 dpi (H) and 9 dpi (J). Data are represented as mean ± SEM from 4 animals in mock, and 5 in infected and 5 in infected+αKG groups at 5 dpi, and 6 animals in infected and 6 in infected+αKG groups at 9 dpi (one‐way ANOVA, using Bonferroni's post‐test, *p < .05, ***p < .001 and ns = non‐significant)

In conclusion, our study describes that αKG significantly inhibits SARS CoV‐2 replication and reduces inflammation, thrombosis and apoptotic cell death in the lungs to restore normal SpO_2_ saturation in infected animals without affecting the anti‐viral response of CD4 and CD8 T cells, and IgG. Therefore, our study strongly suggests a potential use of this metabolite as one of the first‐line therapeutics for COVID‐19. It may be used alone or in combination with current drugs for the disease. The employment of αKG may open up new avenues of treatment for lung inflammation and thrombosis in other respiratory diseases as well.

## COMPETING INTERESTS

The authors declare that they have no competing interests.

## Supporting information

Supporting information
