# Supplementary material for: Dietary alpha‐ketoglutarate inhibits SARS CoV‐2 infection and rescues inflamed lungs to restore O2 saturation by inhibiting pAkt
Source: Clin Transl Med. 2022 Sep 19;12(9):e1041. doi: 10.1002/ctm2.1041 (PMC9484267; doi:10.1002/ctm2.1041)
Supplement: Supplementary file 1 — Supporting information [file CTM2-12-0-s001.pdf]

Online Supporting Information

Dietary alpha-ketoglutarate inhibits SARS CoV-2 infection and rescues inflamed lungs to restore O2 saturation by inhibiting pAkt

Sakshi Agarwal[1,$], Simrandeep Kaur[1,$], Tejeswara Rao Asuru[1,#], Garima Joshi[1,#], Nishith M Shrimali[1], Anamika Singh[1], Oinam Ningthemmani Singh[2], Puneet Srivastva[2], Tripti Shrivastava[2], Sudhanshu Vrati[1], Milan Surjit[2], Prasenjit Guchhait[1,*].

[1]Regional Centre for Biotechnology, National Capital Region Biotech Science Cluster, Faridabad, India; [2]Translational Health Science Technology Institute, National Capital Region Biotech Science Cluster, Faridabad, India. *Correspondence at prasenjit@rcb.res.in

Supplemental Figures

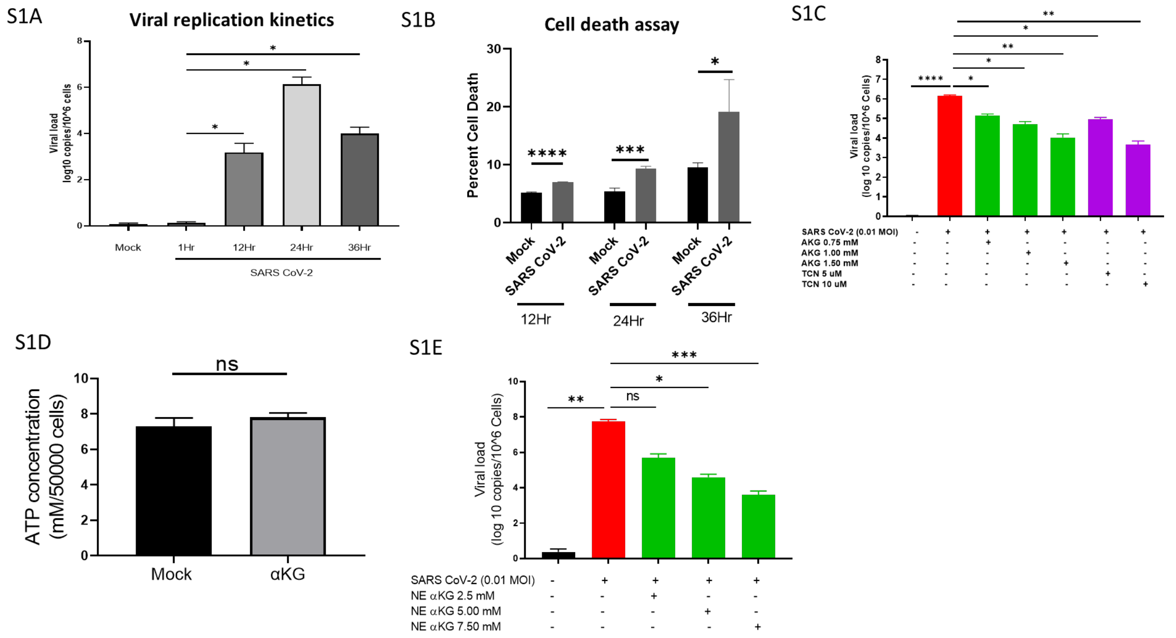

Figure S1. Viral replication kinetics and cell death assay, is decreased by αKG supplementation: (A) SARS CoV-2 replication at 1hr, 12hr, 36hr time points using RT-PCR in Vero E6 cells. Data are mean ± SEM from 3 independent experiments. (B) Cell death assay using Propidium iodide (100ug/ul) for staining for same time points as mention above. Data are mean ± SEM from 3 independent experiments. Unpaired t-test with post Mann-Whitney test (*P<0.05). (C) Viral load in Vero E6 cells infected with SARS CoV-2 (0.01 MOI) for 24 hr in presence of Octyl- αKG or TCN. Data are from 6 independent experiments and represented as mean ± SEM (Kruskal-Wallis test followed by Dunn's multiple comparison post-test), *P<0.05, **P<0.01, ***P<0.001, and ns=non-significant. (D) ATP measurement in Vero E6 cells 24 hours post supplementation with αKG. Data are mean ± SEM from 3 independent experiments. (E) Viral load in Vero E6 cells infected with SARS CoV-2 (0.01 MOI) for 24 hr in presence of non-esterified αKG. Data are from 3 independent experiments and represented as mean ± SEM (Kruskal-Wallis test followed by Dunn's multiple comparison post-test), *P<0.05, **P<0.01, ***P<0.001, and ns=non-significant.

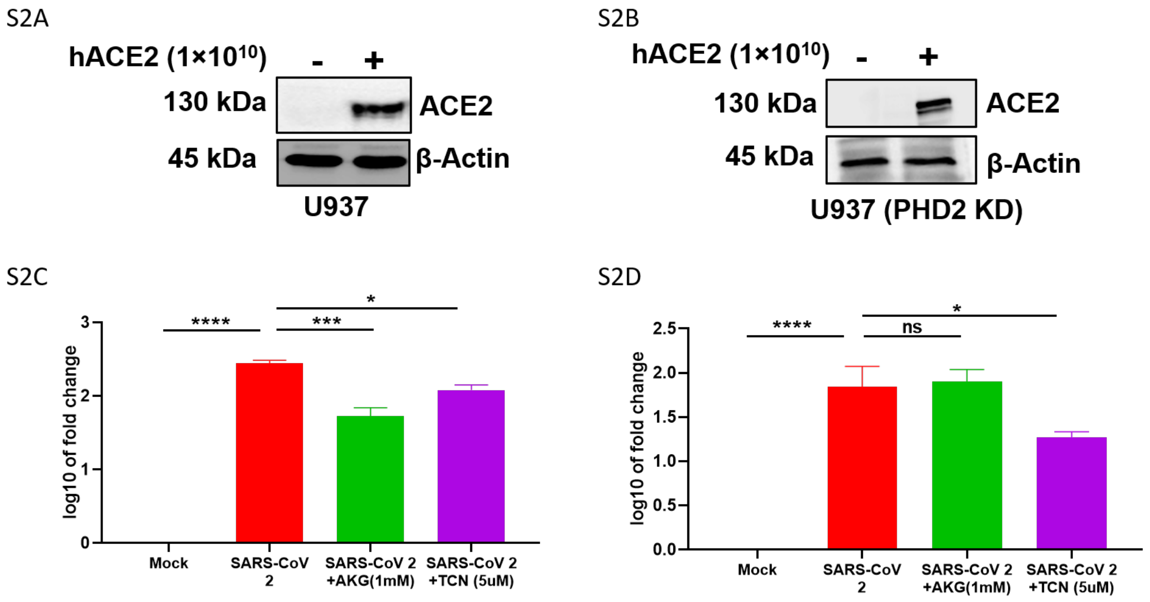

Figure S2: αKG inhibits pAkt via PHD2 axis: (A-B) ACE2 was expressed transiently in (A) monocytic U937 cell line or (B) PHD2-knockdown U937 cells using a human ACE2 adenovirus construct. (C) αKG or TCN decreased SARS CoV-2 viral replication (negative strand of viral RNA measured in wild-type U937 cells). (D) αKG was unable to decrease viral replication (negative strand of viral RNA in PHD2-knockdown U937 cells). TCN decreased viral replication in PHD2-KD U937 cells. Data are from three independent experiments and represented as mean ± SEM (one-way ANOVA using Kruskal-Wallis's post-test), *P<0.05, ***P<0.001, ****P<0.0001 and ns=non-significant.

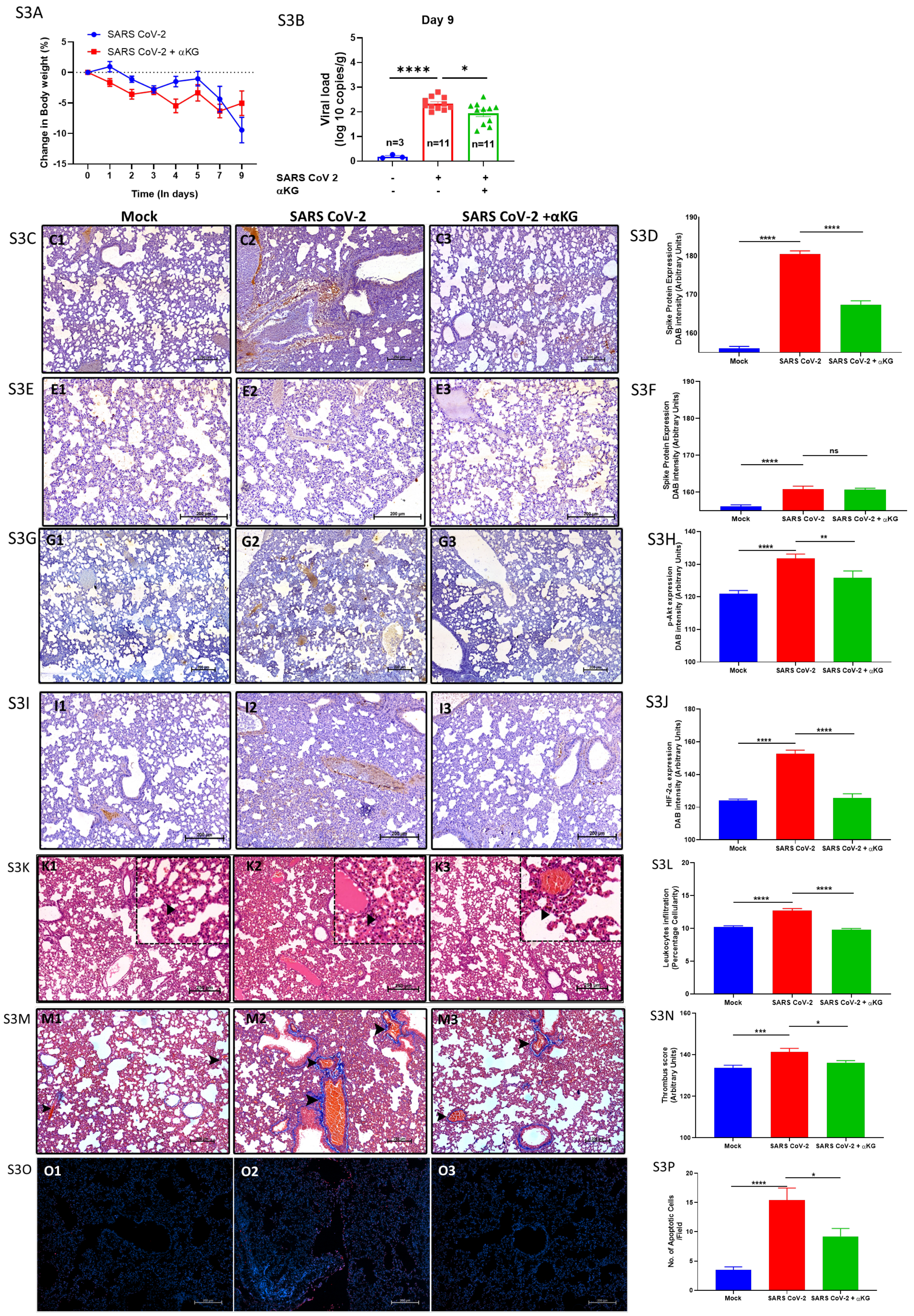
S3A
SARS CoV-2
SARS CoV-2 + αKG
Change in Body weight (%)
Time (In days)
S3B
Day 9
Viral load (log 10 copies/g)
n=3
n=11
n=11
SARS CoV 2
αKG
Mock
SARS CoV-2
SARS CoV-2 +αKG
S3C
C1
C2
C3
S3D
Spike Protein Expression DAB intensity (Arbitrary Units)
S3E
E1
E2
E3
S3F
S3G
G1
G2
G3
S3H
p-Akt expression DAB intensity (Arbitrary Units)
S3I
I1
I2
I3
S3J
HIF-2α expression DAB intensity (Arbitrary Units)
S3K
K1
K2
K3
S3L
Leukocytes infiltration (Percentage Cellularity)
S3M
M1
M2
M3
S3N
Thrombus score (Arbitrary Units)
S3O
O1
O2
O3
S3P
No. of Apoptotic Cells /Field

Figure S3: αKG rescues hamsters from SARS CoV-2 infection: (A) Body weight change in hamsters after SARS CoV-2 infection with and without αKG supplementation. (B) αKG reduces the SARS CoV-2 viral replication in hamster lung (measured by RT-PCR at 9 dpi). Data are from 3 mock and 11 each infected and infected+αKG at 9 dpi. Each dot represents individual value. Data are represented as mean ± SEM (one-way ANOVA using Sidak's post-test), **P<0.01 and ****P<0.0001. (C-F) Expression of Spike protein in hamster lungs was reduced upon αKG supplementation at 5 dpi (3C) and 9 dpi (3E) measured by IHC. DAB intensity was quantified at 5 dpi (3D) and 9 dpi (3F). Data are from 10 fields from different animals. Values are represented as mean ± SEM (one-way ANOVA using Bonferroni's post-test), ****P<0.0001 and ns=non-significant. (G) pAkt expression in αKG treated SARS CoV-2 infected lung measured using IHC. (I) HIF2α expression in αKG treated SARS CoV-2 infected lung measured using IHC at 5 dpi. (H, J) DAB intensity quantification of pAkt and HIF2α expression. Data are from 10 fields from different animals. Values are represented as mean + SEM (one-way ANOVA using Bonferroni's post-test), **P<0.01 and ****P<0.0001. (K) H&E staining of lung was used to assess leukocyte accumulation at 9 dpi. Score was calculated as percentage cellularity at 9dpi (L), arrows indicate cell accumulation. Scale bar 250 mm. αKG supplementation reduced inflammation in infected hamsters. Data are from 10 fields from different animals. Values are represented as mean ± SEM (one-way ANOVA, using Bonferroni's post-test **** P<0.0001). (M) MT staining of lung was used for assessing clot formation at and 9 dpi, arrows indicate thrombus or clot. Score was calculated using color deconvolution protocol in image-J software at 9 dpi (N). Scale bar 200 mm. αKG treatment reduced thrombosis in infected hamsters. Data are from 10 fields from different animals. Values are represented as mean ± SEM (one-way ANOVA, using Bonferroni's post-test *P<0.05, ***P<0.001 and **** P<0.0001). (O) TUNEL assay in the lungs of infected hamsters was used to assess apoptotic cells (red puncta). αKG treatment reduced apoptosis in the lungs of infected hamsters at 9 dpi. (P) TUNEL assay quantification as number of cells per field. 3X3 field captured at 20X magnification. Data are from 10 fields from different animals and represented as mean ± SEM (one-way ANOVA, using Bonferroni's post-test *P<0.05 and **** P<0.0001)

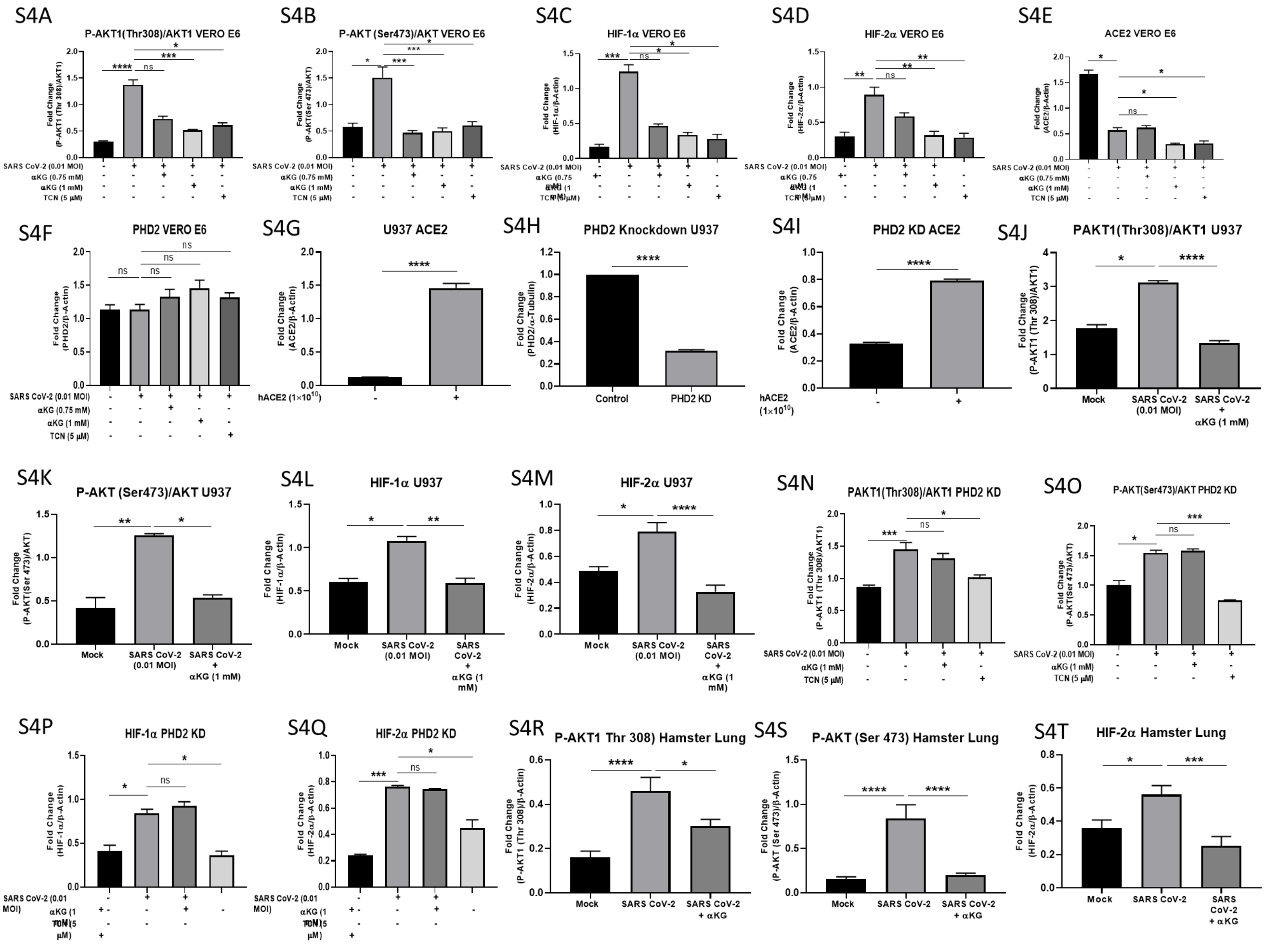

Figure S4. Densitometry of western blots. Densitometric evaluation of the protein bands was performed using the Alpha imager software for western blots. Densitometry related to western depicted in main Figure 1C (Figure S4A-F), Figure S2A (Figure S4G), Figure 1I (Figure S4H), Figure S2B (Figures S4I), Figure 1H (Figures S4J-M), Figure 1J (Figures S4N-Q), Figure 2I (Figures S4R-T). Bar represents mean ± SEM from triplicate blots. Kruskal Wallis test used to compare between the groups, **P*<0.05, ***P*<0.01, ****P*<0.001, **** *P*<0.0001, ns=non-significant.

S5

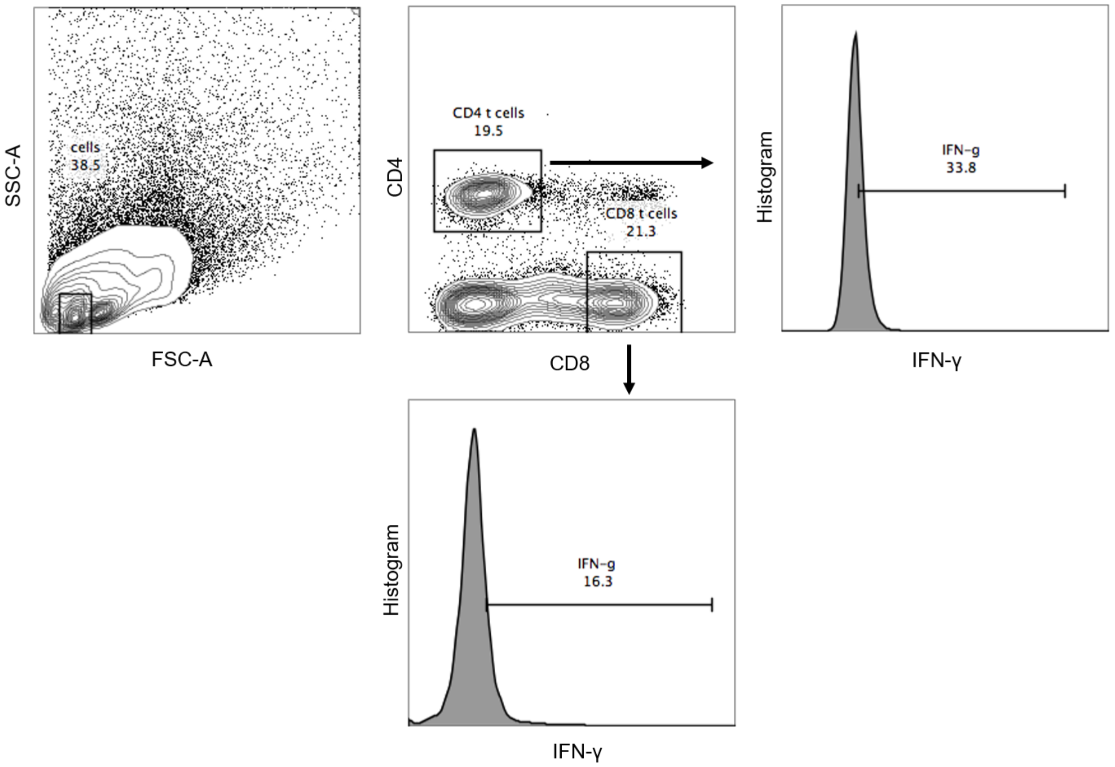

Figure S5. Gating strategy for interferon-γ positive (IFNγ+) CD4+ and IFNγ+CD8+ T cells.

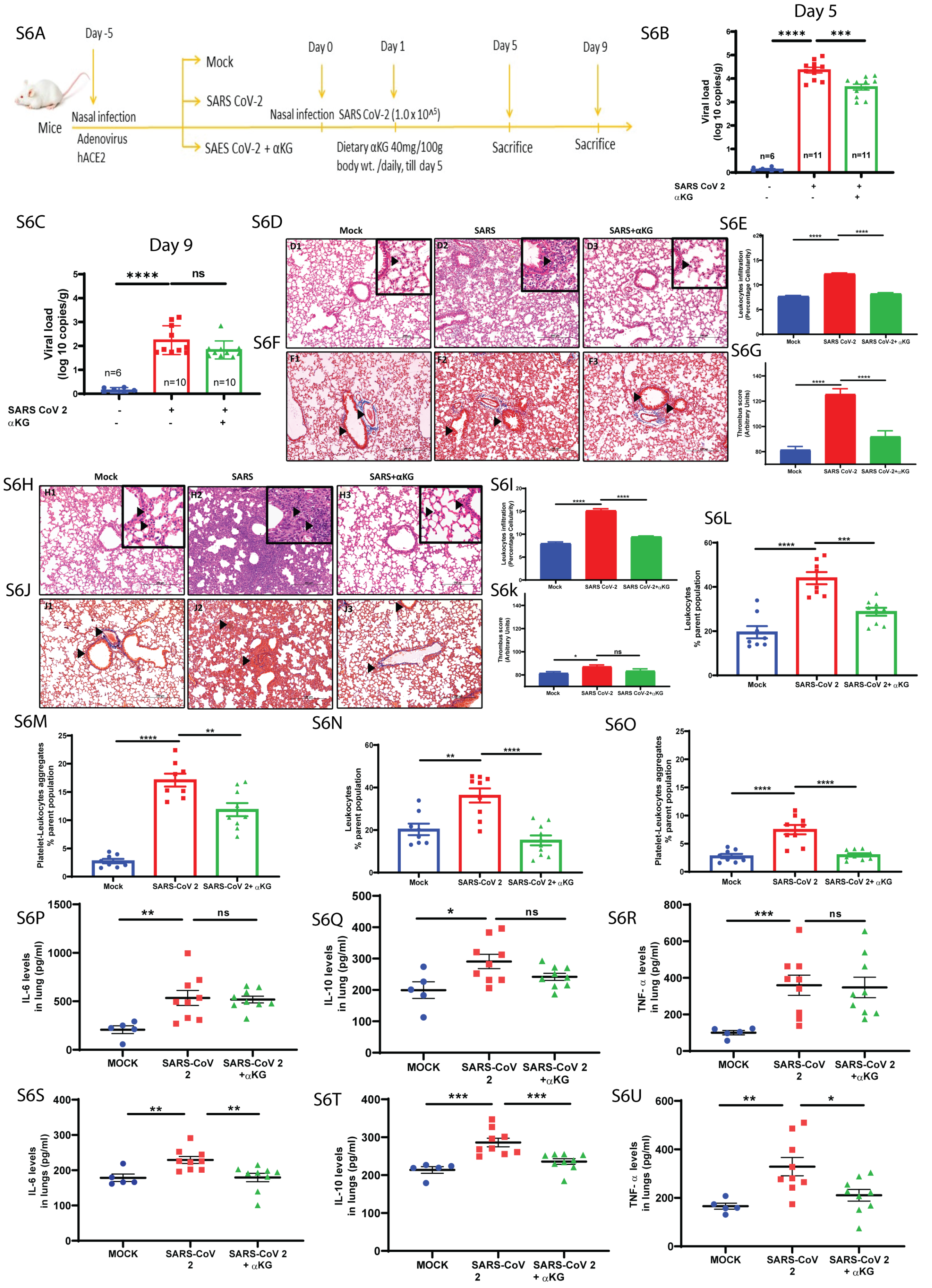
S6A
Day -5
Mock
Day 0
Day 1
Day 5
Day 9
SARS CoV-2
Nasal infection
Adenovirus
hACE2
Mice
Nasal infection SARS CoV-2 (1.0 x 10^5)
SAES CoV-2 + αKG
Dietary αKG 40mg/100g body wt. /daily, till day 5
Sacrifice
Sacrifice
S6B
Day 5
Viral load (log 10 copies/g)
n=6
n=11
n=11
SARS CoV 2
αKG
S6C
Day 9
Viral load (log 10 copies/g)
n=6
n=10
n=10
SARS CoV 2
αKG
S6D
Mock
SARS
SARS+αKG
S6E
Leukocytes infiltration (Percentage Cellularity)
Mock
SARS CoV-2
SARS CoV-2+ αKG
S6F
S6G
Thrombus score (Arbitrary Units)
S6H
Mock
SARS
SARS+αKG
S6I
Leukocytes infiltration (Percentage Cellularity)
S6J
S6k
Thrombus score (Arbitrary Units)
S6L
Leukocytes % parent population
Mock
SARS-CoV 2
SARS-CoV 2+ αKG
S6M
Platelet-Leukocytes aggregates % parent population
S6N
Leukocytes % parent population
S6O
Platelet-Leukocytes aggregates % parent population
S6P
IL-6 levels in lung (pg/ml)
MOCK
SARS-CoV 2
SARS-CoV 2 +αKG
S6Q
IL-10 levels in lung (pg/ml)
S6R
TNF- α levels in lung (pg/ml)
S6S
IL-6 levels in lungs (pg/ml)
S6T
IL-10 levels in lungs (pg/ml)
S6U
TNF- α levels in lungs (pg/ml)

Figure S6: **αKG inhibits inflammation and thrombosis in the lungs of SARS CoV-2 infected mice**. (A) Schematic representation of experimental protocol. Balb/c mice were given human ACE2 adenovirus construct via intra nasal route for transient expression of ACE2, after 5 days mice were given SARS CoV-2 infection via nasal route and αKG was supplemented from 1 dpi. Mice were sacrificed at 5 dpi and 9 dpi and following parameters were measured. (B-C) αKG reduces the SARS CoV-2 viral replication in mice (measured by RT-PCR, 5 dpi and 9 dpi) Data are from 6 mock and 11 each from infected and infected+αKG group at 5 dpi and from 6 mock and 10 each from infected and infected+αKG group at 9 dpi. Values are represented as mean ± SEM (one-way ANOVA using Sidak's post-test), ***P<0.001, ****P<0.0001 and ns=non-significant. (D-K) (D-E, H-I) H&E staining of lung was used for assessing leukocyte accumulation 5 dpi (D) and 9 dpi (H). Score was calculated as percentage cellularity at 5 dpi (E) and 9 dpi (I), showing a reduced inflammation by αKG treatment. Data are from 10 fields from different animals. Values are represented as mean ± SEM (one-way ANOVA, using Bonferroni's post-test **** P<0.0001). (F-G, J-K) MT staining of lung was used to assess thrombus score at 5 dpi (F) and 9 dpi (J). Score was calculated using color deconvolution protocol in image-J software at 5 dpi (G) and 9 dpi (K), showing reduced thrombosis in αKG-treated hamsters. Data are from 10 fields from different animals. Values are represented as mean ± SEM (one-way ANOVA, using Bonferroni's post-test *P<0.05, **** P<0.0001 and ns=non-significant). (L-O) Leukocytes population and leukocyte-platelet aggregation population in single cell suspension of lung was assessed using flow cytometry. αKG treatment reduced leukocytes number in lungs at 5 dpi (L) and 9 dpi (N). αKG treatment also reduced leukocytes-platelet aggregates in lungs at 5 dpi (M) and 9 dpi (O). Data are from 8 animals in mock and 8-10 animals each in SARS CoV-2 infected and αKG-treated. Values are represented as mean ± SEM (one-way ANOVA, using Bonferroni's post-test **P<0.01, ***P<0.001, **** P<0.0001). (P-U) IL6, IL10 and TNFα were measured in lungs of SARS CoV-2 infected animals and αKG-treated animals at 5 dpi (P-R) and 9 dpi (S-U). Data are from 6 animals in mock and 9 animals each in infected and infected+αKG group. Values are represented as mean ± SEM (one-way ANOVA, using Bonferroni's post-test *P<0.05, **P<0.01, ***P<0.001 and ns=non-significant).

S7A

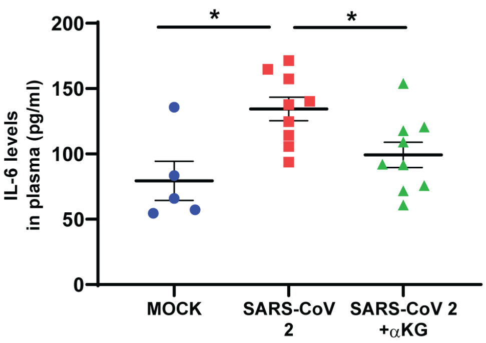

S7B

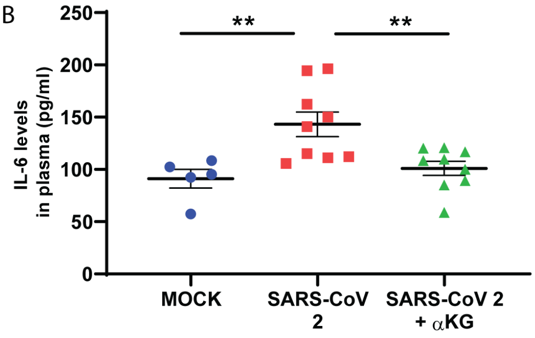

S7C

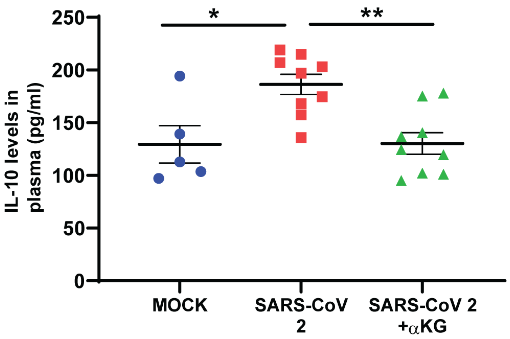

S7D

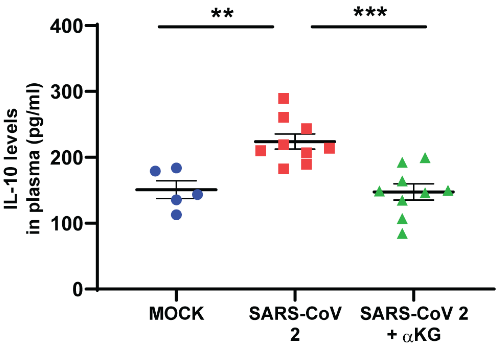

S7E

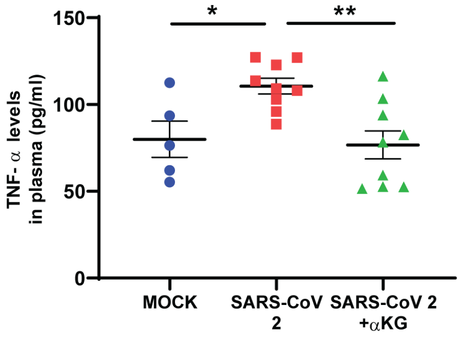

S7F

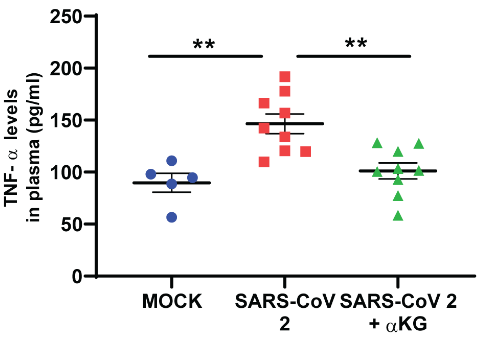

S7G

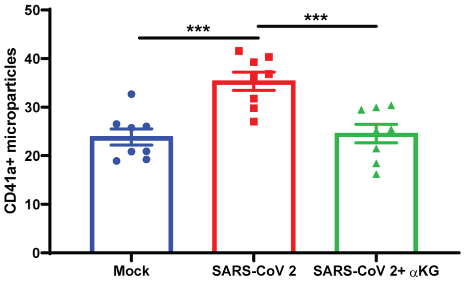

S7H

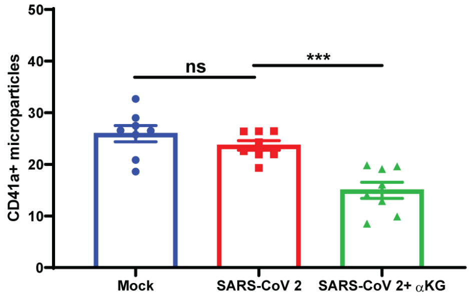

S7I

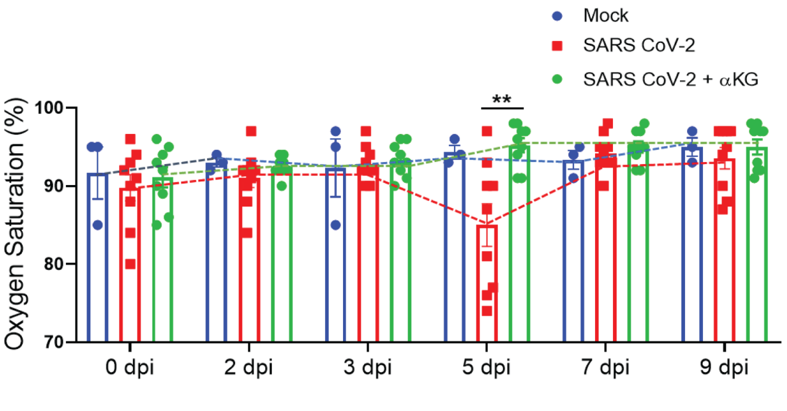

Figure S7: αKG inhibits elevated cytokines and platelet microparticles in SARS CoV-2 infected mice and restores oxygen saturation: (A-F) IL6, IL10 and TNFα were measured in plasma of infected and infected+αKG group at 5 dpi (A-C) and 9 dpi (D-F). αKG reduced above cytokines levels in plasma. Data are from 6 animals in mock and 9 animals each in infected and infected+αKG group. Values are represented as mean ± SEM (one-way ANOVA, using Bonferroni's post-test *P<0.05, **P<0.01, ***P<0.001). (G-H) CD41a+ microparticles were measured using flow cytometry in plasma of SARS CoV-2 infected animals and αKG-treated animals at 5 dpi (G) and 9 dpi (H). αKG reduced CD41a+ microparticles in the plasma. Data are from 8 animals in each group. Values are represented as mean ± SEM (one-way ANOVA, using Bonferroni's post-test, **P<0.01, ***P<0.001 and ns= non-significant). (I) Oxygen saturation was measured in circulation of the infected vs infected+αKG balb/c mice using pulse oximeter. Data are from 9 animals in each group. Values are represented as mean ± SEM (student's t test, *P<0.05 and ns=non-significant).

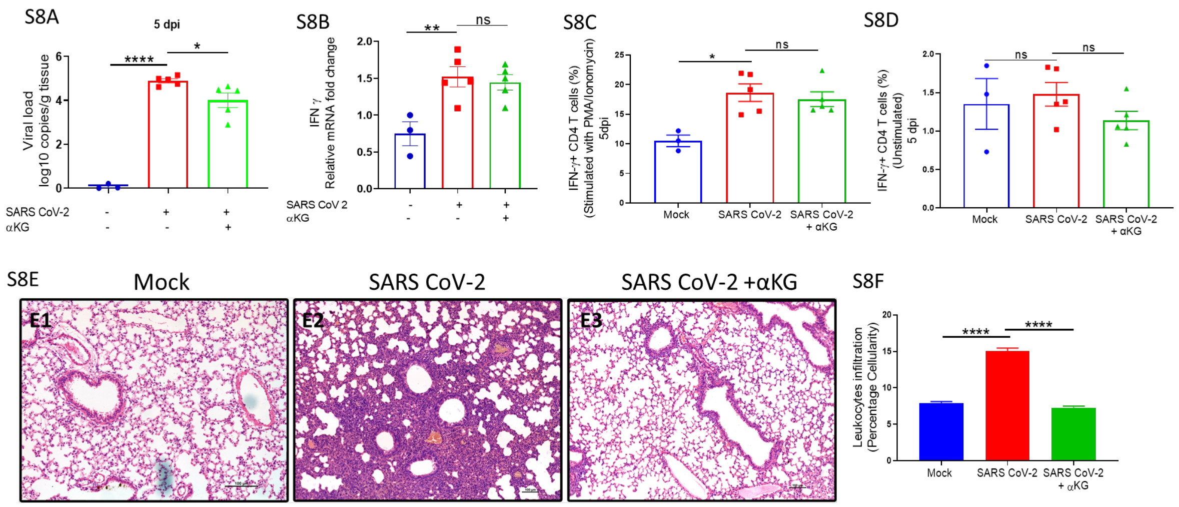

Figure 8: Female mice were infected with SARS CoV-2 in the similar manner as described for male mice. (A) Viral load in SARS CoV-2 infected mice with and without αKG supplementation, at 5 dpi. Each dot represents a mouse. Data are the mean ± SEM (One way ANOVA, using Bonferroni's post-test **** P<0.0001 and *P<0.05. (B) IFN-γ relative mRNA expression from spleen of infected and infected+αKG animals. Data are the mean ± SEM (One way ANOVA, using Bonferroni's post-test **P<0.01 and ns=non-significant. (C) IFNγ+ CD4 T cells stimulated with PMA/ionomycin and, (D) without stimulation from spleen of infected and infected+αKG groups at 5 dpi. Without stimulation, very least percentage of IFNγ+ cells were detected. Values are represented as mean ± SEM (Kruskal Wallis test, *P<0.05 and ns=non-significant). (E) H&E staining of lung was used to assess leukocyte accumulation at 5 dpi. Score was calculated as percentage cellularity (F), Data are from 8 fields from different animals and represented as mean ± SEM (one-way ANOVA, using Bonferroni's post-test **** P<0.0001).

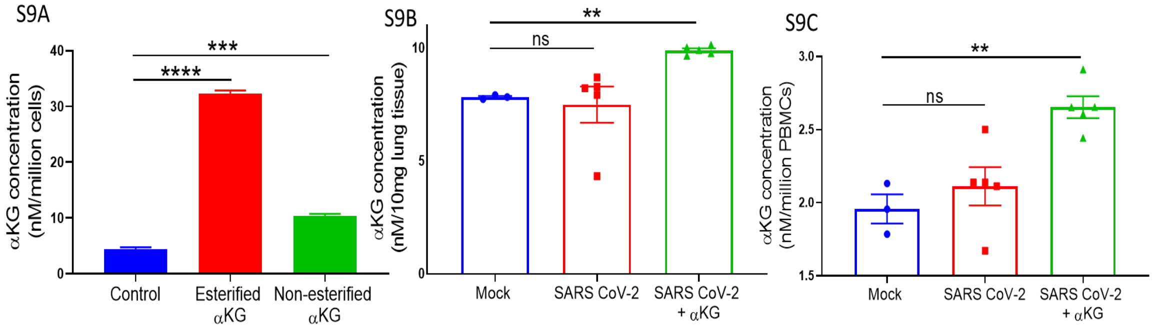

Figure 9: (A) **Intracellular αKG** was measured by kit in Vero E6 cells supplemented with 1mM Octyl αKG and 5mM non-esterified αKG. (B) Intracellular αKG was measured in lung tissue of infected and infected+ αKG mice. (C) Intracellular αKG was measured in PBMCs of infected and infected+αKG mice. All data are represented as mean ± SEM (Kruskal Wallis test) **P<0.01, ***P<0.001 ****P<0.0001, and ns=non-significant.

Supplementary Methods

Ethics

Ethics approval was obtained from the Institutional Animal Ethics Committee (IAEC) (ref. no. RCB/ IAEC/2021/093), and Institutional Biosafety Committee (IBSC; ref. no. RCB/IBSC/21-22/308) of the Regional Centre for Biotechnology (RCB), Faridabad, India. Experiments using BALB/c mouse strain (RRID: IMSR_JAX_000651) and Syrian golden hamster (available form ICMR-National Institute of Nutrition, Hyderabad, India) were conducted within the guidelines of IAEC in the Biosafety level 3 (BSL3) facility of the institute.

SARS CoV-2 infection in Vero and U937 cells

Monkey kidney epithelial cell line Vero E6 ($0.1x10^6$ cells/well) was infected with SARS CoV-2 (world reference # USA-WA-1/2020, as mentioned in our earlier work [3] at 0.01 MOI for 1 hr in Dulbecco's Modified Eagle Medium (DMEM) with 2% FBS, supplemented with 1% non-essential amino acids. One-hour post infection, cells were supplemented with different concentrations of octyl αKG (0, 0.75, 1 and 1.5 mM, Sigma-Aldrich, USA) or non-esterified αKG (SRL Pvt. Ltd., Maharashtra, India) Triciribine (TCN; from Selleckchem, USA) for 24 hr in BSL3 facility. The Vero E6 cell line was validated for free of mycoplasma contamination. Cell pellet was collected in Trizol. Total RNA (1 mg) was reverse-transcribed using iScript select cDNA synthesis kit (Bio-Rad, USA) as per manufacturer's protocol, using primer 5'CGTGTCATGGTGGCGAATAAGATTAAAGGTTTATACCTTCCCAGGTAACA 3' for the synthesis of negative strand cDNA. The cDNA was diluted in nuclease-free water (Promega, USA) and used for real-time PCR with either SARS CoV-2 or 18S rRNA specific primers, using SYBR-green mix (Bio-Rad) in an Applied Biosystems Quant Studio TM 6Flex Real-Time PCR System. The oligonucleotides used were SARS-F (5' GTGTCATGGTGGCGAATAAG 3') and SARS-R (5' TCGTTGAAACCAGGGACAAGG 3')

for SARS-CoV-2, and 18S r-RNA-F (5' GGCCCTGTAATTGGAATGAGTC 3') and 18S rRNA (5'CCAAGATCCAACTACGAGCTT3'). The Ct value corresponding the viral RNA was normalised to that of 18S rRNA transcript. The relative level of SARS CoV-2 RNA in mock-infected samples was arbitrarily taken as 1 and that of infected samples expressed as fold-enrichment (FE). The FE was calculated as 10 logarithmic value and plotted.

SARS CoV-2 infection in U937 cells: Wild type U937 monocytic cells line or PHD2-knockdown U937 cells (80-90% knockdown efficiency) were transfected with a human ACE2 adenovirus (AD5CMVACE2, Viral Vector Core Web, University of Iowa, USA) with a concentration of 10 MOI for 1 hr in the presence of 8 μm polybrene. After wash with PBS, cells were kept in fresh media having 2% serum for 48 hr, then we proceeded with the SARS CoV-2 infection at 0.01 MOI for 1 hr and wash the cells and kept in fresh media for 24 hr with and without octyl αKG (1 mM as mentioned in our previous work [3]) or TCN (5 μM, this concentration was found to be effectively inhibiting pAkt in U937 cells, our unpublished work) treatment.

SARS CoV-2 infection in hamster and mice

We used Syrian golden hamster model of SARS CoV-2 and a modified protocol of infection as described in our previous work [3]. Male hamsters of 9-10 weeks old were infected with one-time SARS CoV-2 via nasal route inoculation using $1x10^5$ plaque-forming units (PFU). Animals were supplemented with a human dietary grade αKG (Double Wood LLC, Philadelphia, USA) with a dose of 40 mg/100 g body wt. (dissolved in water), through oral gavage from day 1 to day 4 daily post infection. At day 5 and day 9 post infection, animals were sacrificed and blood, lung and spleen samples were harvested. Details are described in schematic Figure 2A.

The balb/c, 9-10 weeks old male and female mice were treated (one-time through intranasal route) with human ACE2 adenovirus (described above) using $25x10^8$ PFU for transient expression of hACE2 in respiratory track. Mice were infected with SARS CoV-2 via nasal route inoculation using $1x10^5$ PFU as described [9]. Animals were supplemented with αKG with a dose of 8 mg/20 g body wt., through oral gavage from day 1 to 4 post infection. Animals were sacrificed at day 5 and day 9 post infection. Blood and lungs were collected from the animals. Details are described in schematic Figure S6A.

PCR estimation of viral genome from lung tissue

Lung tissue sample from hamsters and mice was homogenized in Trizol using a hand-held tissue homogenizer and the total RNA extracted as per manufacturer's protocol. RNA was reverse-transcribed using iScript™ cDNA synthesis kit (Bio-Rad) as per manufacturer's protocol. The cDNA was used for real-time PCR for SARS CoV-2 or G3PDH, as described above. The primer used were SARS SN1-F (5'-GACCCCAAAATCAGCGAAAT -3') and SARS SN1-R (5'TCTGGTTACTGCCAGTTGAATCTG-3') for SARS CoV-2, and G3PDH-F (5'-GACATCAAGAAGGTGGTGAAGCA-3') and G3PDH-R (5'-CATCAAAGGTGGAAGAGTGGGA-3'). The Ct value corresponding the viral RNA was normalised to that of G3PDH transcript. The relative level of SARS CoV-2 RNA in mock

samples was arbitrarily taken as 1 and that of infected samples expressed as fold-enrichment, as described in our previous work [3].

Confocal microscopy

Vero E6, U937 cells or U937/PHD2-KD cells from above experiment were fixed in 4% paraformaldehyde for 20 min at room temperature. After wash with 1xPBS, cells were permeabilized using 0.1% Triton X-100 followed by 2 hr blocking with 5% BSA and 5% FBS at RT. The cells were labelled with primary antibodies keep it for overnight followed by Alexa 594 conjugated secondary antibody. Further, they were stained with DAPI for 10 min and then cells were mounted with ProLong Gold anti-fade reagent. Images were captured using a Leica Confocal DMI 6000 TCS-SP8 microscope (Leica Microsystems, Wetzlar, Germany) at 63× oil immersion objective (NA 1.4) Plan Apo objectives and quantified as mentioned. Imaging was performed using Z-stacks at 0.25 μm per slice by sequential scanning and Image J Fiji software was used to obtain maximum intensity projection images. The total cell fluorescence intensity was calculated from at least 25 cells in each set per experiment and data represented as mean and standard error of mean from at least 3 independent experiments.

Western blotting

The lysate of cell pellet or lung tissue was prepared in RIPA lysis buffer with protease-phosphatase inhibitor (Thermo Scientific Life Tech, USA) at BSL3 facility. The lysate samples were further processed for SDS-PAGE followed by immunoblotting using primary antibodies against pAkt-Ser473, Akt, pAkt1-Thr308, Akt1, HIF1α, HIF2α, β-Actin (Cell Signalling, USA), as described in our work [3].

Immunohistochemistry, microscopy

Lungs from hamsters and mice were fixed in 10% formalin solution and processed for paraffin embedding. 3 μm thick sections of the embedded tissues were cut for staining. All samples were stained with hematoxylin and eosin; and Masson's trichrome stains. Tissue sections from hamsters were stained for pAkt, HIF2α and Spike proteins in the lungs. Each stained section was analysed and captured at 20X magnification. Cellularity, Thrombosis score and intensity of Immunohistochemistry were quantified using ImageJ software. Cellularity was calculated as percentage of area covered by the nucleus as described in our work [9] Thrombosis and immunohistochemistry were quantified using colour deconvolution method in the software.

Immunophenotyping

Single cell suspension was prepared from lung tissue using Liberase (100 μg/ml; Sigma-Aldrich). Chopped lung pieces were kept in Liberase for 1 hr at 37° C. After the lung chunks started to dissolve in the form of cell suspension, cells were strained using cell strainer and washed. After RBC lysis PBMCs were stained for measuring leukocytes and leukocytes-platelet aggregates. Cells were stained with anti-mouse CD45.2 APC and CD41a PE (BD Biosciences, San Jose, USA) for 30 min in dark at RT. Cells were then washed and acquired on BD FACS Verse and were analyzed with FlowJo software (Tree Star, USA).

Cytokine assay
Cytokines such as TNFα, IL6 and IL10 were measured from mice plasma of different treatments at different day points using CBA and analysed by FCAP array software (BD Biosciences, USA).

Platelet microparticle
Platelet poor plasma (PPP) was obtained by centrifuging Platelet rich plasma at 1000 g for 10 min. Platelet free plasma was obtained from PPP in 2 sequential steps: PPP at 2500 g for 15 min, and again at 5000 rpm for 15 min. The protocol is modified according to mouse plasma. Platelet-derived microparticles were measured using flow cytometry after labelling with anti-mouse CD41 PE (BD Biosciences, USA) as described in our work [3].

Tunel assay
Assay was performed according to manufacturer's protocol. Paraffin-embedded tissue sections were dewaxed and rehydrated using xylene and ethanol gradient. Tissues sections were then microwave irradiated in 0.1 M citrate buffer, pH 6.0 for 10 min. 50 µl TUNEL reaction mixture was homogeneously spread over the tissue section and incubated in a humidified atmosphere for 60 min at 37° C in the dark. Sections were washed and mounted with mounting media prolong gold. Image was captured in fluorescence microscopy with an excitation wavelength in the range of 520-560 nm and detection in the range of 570-620 nm.

SARS CoV-2 RBD antibody measurement
The binding-antibody response to SARS CoV-2 post-infection was measured using ELISA based platform as described earlier [10]. The antibody response was measured against RBD protein post virus challenge at different time points. Soluble RBD protein was coated on Maxisorp plates (Nunc) with 2µg/ml of purified protein and incubated with three-fold serially diluted sera (with 1:5 as starting dilution) in dilution buffer (1:5 times dilution of blocking buffer). The plates were incubated at room temperature (RT) for 1 h and then washed three times with washing buffer (PBS + 0.1 % tween 20) and incubated with HRP conjugated anti-hamster IgG antibody at 1:10,000 dilution for another 1 h and washed four times with the washing buffer and incubated further with 100 µl of TMB substrate (Thermo Fisher Scientific), The reaction stopped with 1N H_2SO_4 solution. Sera end point titer were calculated as the reciprocal of serum dilution giving OD 450 nm readings higher than lowest dilution of the placebo or control arm + two times standard deviations.

T cell activation assay
Splenocytes were isolated from the hamster spleen. Spleen was crushed using frosted slide and single cell suspension was prepared using wire mesh. After RBC lysis from single cell suspension, splenocytes (1 million cells from each animal) was stimulated with PMA (phorbol 12-myristate13-acetate; 50 ng/ml; Sigma-Aldrich), ionomycin (1.0 µg/ml; Sigma-Aldrich) for 6 hr. Cell surface staining with anti-mouse CD4 PE and CD8 APC (BD Biosciences, USA) was carried out for 30 min in dark at RT. Cells were then fixed in IC fixation buffer (Thermo Fisher Scientific, USA) for 30 min each. Intracellular anti-mouse IFN-γ APC Cy7 (BD

Biosciences, USA) staining was then carried out for 30 min in dark at RT. Cells were acquired and analysed as described above.

Neutralizing antibody measurement

Vero E6 cells were maintained in DMEM medium supplemented with 10% FBS, 100 U/ml of penicillin–streptomycin and 1% non-essential amino acids. The assay was performed in duplicate using 24-well tissue culture plates, in a biosafety level 3 facility. Serial dilutions of each plasma sample were incubated with 30-40 PFU of virus for 1 hr at 37°C. The virus-serum mixtures were added to Vero E6 cell monolayers and incubated for 1 hr at 37°C in 5% CO_2 incubator. The cell monolayer was then overlaid with 2% CMC in cell culture medium and incubated for 2 days, at which time the plates were fixed in 3.7% formaldehyde and stained with crystal violet dye. $PRNT_{50}$ represents antibody concentration at which 50% reduction in plaque formation occurs. $PRNT_{50}$ was calculated with the help of non-linear curve using GraphPad Prism software.

SpO_2 measurement

Oxygen saturation (SpO_2) in the blood was measured in both mice and hamsters from the above experiments. The Mouse STAT Jr. pulse oximeter by Kent Scientific Corporation, Torrington, USA was used for the measurement of SpO_2 in awake animals. Paw of the animals was captured in the sensor.

ATP measurement

Vero E6 cells were seeded in DMEM medium at the density of 50,000 cells per well. They were treated with and without octyl alpha ketoglutarate for 24 hours. ATP was measured by Cell Titer-Glo kit (Promega, USA). Cell Titer-Glo reagent was added to the cells, mixed and kept for 10 minutes at orbital shaker to induce cell lysis. Incubated plate at room temperature for 10 minutes to stabilize luminescent signal. Luminescence was recorded and compared with the standard curve generated from ATP disodium salt (Sigma, cat no. A2333).

αKG measurement

αKG level was measured in cell lysate using colorimetry based assay kit (Sigma Aldrich, USA, catalog No. MAK054 and MAK335) according to the manufacturers' protocol, as described in our previous works [3,4].

Statistical analysis

Data from at least three experiments are presented as mean ± SEM (standard error of the mean). Statistical differences among experimental sets with normally distributed data were analyzed by using one-way or two-way ANOVA followed by Bonferroni's correction for multiple comparison. Kruskal Wallis test followed by Dunn's multiple comparison post-test was used for non-normally distributed data. D'Agostino-Pearson Test was used to check for normal distribution of data. Graph Pad Prism version 8.0 software was used for data analysis and P-values.
